# Barriers and facilitators for screening and treatment of hyperlipidemia among patients with inflammatory arthritis

**DOI:** 10.1186/s41927-020-00123-w

**Published:** 2020-06-02

**Authors:** Iris Navarro-Millán, Sarah R. Young, Sally Shurbaji, Chastity McDavid, Anna Cornelius-Schecter, Bernadette Johnson, Andrea L. Cherrington, Liana Fraenkel, Susan M. Goodman, Jeffrey R. Curtis, Shilpa Venkatachalam, Monika M. Safford

**Affiliations:** 1 Weill Department of Medicine, Division of General Internal Medicine, Weill Cornell Medicine, 420 East 70yth Street – LH -363, New York, NY 10021 USA; 2 Division of Rheumatology, Hospital for Special Surgery, 535 East 70yth Street – LH -363, New York, NY 10021 USA; 3 Department of Social Work, Binghamton University, Binghamton, NY USA; 4 Division of Infectious Diseases, Department of Medicine, University of Alabama at Birmingham, Birmingham, AL USA; 5 Division of Preventive Medicine, Department of Medicine, University of Alabama at Birmingham, Birmingham, AL USA; 6 Yale University, New Haven, USA; 7 Berkshire Health Systems, Pittsfield, MA USA; 8 Division of Clinical Immunology and Rheumatology, Department of Medicine, University of Alabama at Birmingham, Birmingham, AL USA; 9 Global Healthy Living Foundation, Upper Nyack, New York USA

**Keywords:** Cardiovascular disease, Rheumatoid arthritis, Psoriatic arthritis, Hyperlipidemia, Peer coaches, Social support, Patient activation

## Abstract

**Background:**

Patients with inflammatory arthritis (IA), defined as rheumatoid arthritis (RA) and psoriatic arthritis (PsA), are at increased risk for cardiovascular disease (CVD). The frequency of screening and treatment of hyperlipidemia, a modifiable CVD risk factor, is low in these patients. The reasons for low screening and treatment rates in this population are poorly understood. Our objective was to elicit the barriers and facilitators for screening and treatment of hyperlipidemia from the perspective of patients with IA.

**Methods:**

We conducted a qualitative study using focus groups of patients with IA, guided by Bandura’s Social Cognitive Theory. We recruited patients with IA aged 40 years and older from a single academic center. Data were analyzed thematically.

**Results:**

We conducted three focus groups with 17 participants whose mean age was 56 (range 45–81) years; 15 were women. Four themes emerged as barriers: 1) need for more information about arthritis, prognosis, and IA medications prior to discussing additional topics like CVD risk; 2) lack of knowledge about how IA increases CVD risk; 3) lifestyle changes to reduce overall CVD risk rather than medications; and 4) the need to improve doctor-patient communication about IA, medications, and CVD risk. One theme emerged as a facilitator: 5) potential for peer coaches (patients with IA who are trained about concepts of CVD risk and IA) to help overcome barriers to screening and treatment of hyperlipidemia to lower CVD risk.

**Conclusion:**

Patients with IA identified educational needs about IA, increased CVD risk in IA and the need for improved doctor-patient communication about screening for hyperlipidemia and its treatment. Patients were receptive to working with peer coaches to facilitate achievement of these goals.

## Background

Cardiovascular disease (CVD) is the most common cause of death among patients with inflammatory arthritis (IA), defined as rheumatoid arthritis (RA) and psoriatic arthritis (PsA) [1–3]. The risk of CVD in patients with IA is reduced when low disease activity and remission is achieved; for example, studies have shown lower rates of myocardial infarction [4] and a decrease CVD morbidity and mortality [5, 6]. In addition, screening and treatment of hyperlipidemia among patients with IA is an important strategy to help reduce CVD risk among patients with IA [7–10]. Despite this knowledge, ours and others’ previous work have shown that a small number of patients with IA are screened for hyperlipidemia [11–13].

Current guidelines for the treatment of cholesterol from the American College of Cardiology (ACC)/American Heart Association (AHA) recommend adults ages 40 to 75 to be evaluated for primary atherosclerotic cardiovascular disease (ASCVD) prevention [14]. The recommendations include having a clinician–patient risk discussion about lifestyle modifications such as having a healthy diet (high intake of vegetables, fruits, whole grains, legumes, low-fat poultry (without the skin), fish/seafood, and nuts, and limits intake of sweets, sugar-sweetened beverages, and red meat) and increasing physical activity before starting statin therapy [14]. To date, most interventions to increase screening and treatment for hyperlipidemia in patients with IA have been focused on changing physician behavior and have been mostly unsuccessful [15, 16]. The lack of success can be attributed to physicians’ alert fatigue from the electronic health record (EHR) system and dissatisfaction with the implementation of CVD risk reduction guidelines within the EHR [15, 17]. Therefore, it opens up a possibility in exploring the effectiveness of an intervention targeting the patient directly about reducing CVD risk.

In conditions such as asthma, human immunodeficiency virus (HIV), and diabetes, peer coaches have been successfully used to improve adherence to self-care recommendations and completing treatment [18–25]. Peer coaches are patients who themselves have the targeted condition (e.g. HIV, diabetes) and have been trained to help others with the same condition in following treatment recommendations from their providers, social support, and improving self-efficacy, which translate into healthy behaviors. Since they are not physicians nor other health professionals, they do not provide any medical advice to the patients they are coaching. Rather, their potential role for patients with IA is to coach other patients with IA on becoming engaged in the shared decision making process regarding primary prevention for CVD with their doctors. To our knowledge, peer coaches have not been used to help patients with IA nor have patients’ perspective on this possible resource been described [26–30].

The objective of this qualitative study was to elicit barriers and facilitators to screening and treatment of hyperlipidemia among patients with IA. Another goal was to identify attitudes towards working with a peer coach for modifiable barriers that can be targeted in future health behavior interventions.

## Methods

### Study design and protocol

We conducted a qualitative study based on Social Cognitive Theory (SCT) [31, 32], to identify the lived experience of how patients with IA understand their CVD risk. The SCT posits three mechanisms of human agency: direct personal agency (self-efficacy), proxy agency (reliance on others acting on one’s behalf, such as parents or partners), and collective agency (coordinated interdependent efforts) [31]. We used these components (defined in Table 1) to inform the topic guide used in the focus groups and to analyze the data. The value of using a theoretical framework is that it helps map the causal relationship between a problem and the factors contributing to it, while identifying modifiable factors that can enable behavioral change. For example, focus group questions related to patient knowledge and motivation corresponded to the “direct personal agency” construct of SCT. Questions related to the use and role of a peer coach to help the patient to get screened and treated for hyperlipidemia related to the “proxy agency” construct of SCT. Questions related to the role of and collaboration with physicians related to the “collective agency” construct of SCT. *Supplement**1* presents the topic guide questions. This study adhered to the Consolidated Criteria for Reporting Qualitative Research (COREQ) [33].

**Table 1:** Social Cognitive Theory Constructs that Informed the Topic Guide and Data Analysis of Focus Groups of Patients with Inflammatory Arthritides

Theoretical Construct	Definition
Self-Efficacy	Perceived ability that one can exercise control over one’s health habits,
Outcome Expectation	The expected costs and benefits for different health habits,
Socio-Cultural Factors	Knowledge of health risks and benefits of different health practices. The perceived facilitators and social and structural impediments to the changes they seek.

The topic guide was reviewed by a multidisciplinary panel of experts in rheumatology, qualitative methods, preventive medicine, social work, health behavior, and a patient with RA. This group also provided feedback and guidance during data analysis. The topic guide focused on eliciting participants’ relationships with their physicians, their knowledge of CVD risk in relation to IA, and barriers and facilitators for screening and treatment of hyperlipidemia. We introduced the concept of a peer coach as a potential facilitator and elicited participants’ perspectives on working with a peer coach. Other questions focused on patient activation and their self-efficacy. The University of Alabama at Birmingham (UAB) and Weill Cornell Medical College (WCMC) Institutional Review Boards approved study procedures. We obtained written informed consent from participants prior to each focus group.

We pilot tested the topic guide during the first focus group by asking participants to provide feedback on the clarity of the session goals and the questions. We modified the topic guide accordingly for the subsequent focus groups. Since the participant responses in the first focus group were similar to those in groups two and three, (total of three focus groups), we included data from the first focus group in the final analysis.

### Participant recruitment and eligibility

Participants were adult volunteers with IA (RA or PsA) recruited from the UAB Rheumatology Clinic from January to October 2016. Participants were purposively sampled to include individuals with IA of age ≥ 40 years, in accordance to the ACC/AHA cholesterol treatment guideline CVD risk evaluation recommendation. Potential subjects were first approached in person and later confirmed their participation by phone. We did not share the results of the study with the participants but one of the investigators in the study is a patient with RA, who provided feedback on the results of the study.

### Focus groups

Research staff trained in qualitative research data collection, moderating focus groups, and qualitative research facilitated each focus group using the topic guide (SS). We conducted focus groups in a private meeting space in the clinical rheumatology building of UAB and all sessions were audio recorded. We asked each participant to complete a questionnaire to collect demographic information and information about health behaviors and medical history before each session. Audio recordings from each session were transcribed verbatim by a medical transcription service. Files were uploaded into NVivo software version 10 (QSR International) for analysis.

### Analysis

Three trained investigators in qualitative research, phenomenology, and social work (INM, SRY, SS) independently review and code data from the first focus group. We used a combination of open thematic coding and a priori coding based on SCT. Thematic coding provides a flexible and open mechanism to condense data into meaningful groups, while a priori coding allows for the streamlining of codes based on anticipated results from Bandura’s theory [31]. After coding the first focus group independently, offering a level of investigator triangulation, the same coders (INM, SRY, SS) met to compare notes and clarify the initial list of codes, that were then discussed with the multidisciplinary team that vetted the topic guide for feedback [34]. The coders then independently analyzed the next two focus groups based on the consensus codebook and met again to reconcile any issues. At this meeting, the team developed a final codebook, combining all themes after we reached consensus. We achieved theme saturation, that is, no new information emerged, in the coding of the transcript of the second focus group. We coded the third focus group to ensure rigor and, after coding the data, we confirmed that saturation had been reached because no new information emerged. One coder (SRY) then took the final codebook and re-coded all transcripts. The team met for a final time to review the re-coded data to ensure accuracy.

## Results

The three focus groups included 17 patients with IA; two groups had six participants each and one group had five participants. The mean age of participants was 56 years (range 45–81). Among all participants, 15 had RA and two had PsA, 15 were women (10 Blacks, 5 Whites) and two were White males. Twelve expressed having had a cholesterol test, three were on a statin, one had history of stroke, 12 were on methotrexate, three were on adalimumab, one was on certolizumab, and one was on tofacitinib. Six participants had lived with IA for ≤10 years and seven for > 10 years. Seven of eleven participants indicated on the pre-focus group questionnaire that they were aware that patients with IA have a higher risk for CVD than the general population.

Five overarching themes emerged from the focus groups: four barrier themes and one facilitator theme. Table 2 shows the themes within each construct of SCT.

**Table 2:** Themes and Key Points That Emerged from Focus Groups with Patients with Inflammatory Arthritis (IA) about Cardiovascular Disease (CVD)

Themes	Social Cognitive Theory Construct	Key Points
**Barrier Themes**		
**Theme 1:*Need for more information about arthritis, prognosis, and IA medications prior to discussing additional topics like CVD risk***	Socio-cultural Factors (Limited resources to learn about IA and limited knowledge about IA itself)	Participants were interested in understanding the following: o The expectations for living with arthritis o Side effects of arthritis medication and possible interaction with other medications o Risks and benefits of being on medications for arthritis o Interest in learning about healthy behaviors that they can do to avoid an arthritis flare (ex. exercise or avoid certain types of food)
**Theme 2*: Lack of knowledge about how IA increases cardiovascular disease (CVD) risk***	Socio-cultural Factors (Limited knowledge about the relationship between IA and CVD)	Participants were interested in learning about the following: o How arthritis affects the heart o How they can decrease CVD risk (exercise, diet, stress reduction, and medications) o Side effects of lipid lowering medication and interactions with other medications o Learning about their increased CVD risk associated with IA resulted in some participants feeling motivated to request a cholesterol test
**Theme 3:*Lifestyle changes to reduce overall CVD risk rather than medications***	Self-efficacy	Participants were interested in the following: o Integrating CVD risk reduction program within the overall arthritis management program, and not addressing CVD as a separate issue o Learning about engaging in lifestyle changes to control cholesterol before considering initiation of a statin
**Theme 4:*Improving doctor-patient communication about IA, medications, and CVD risk***	Social Support Outcome expectation	Participants considered their treating physician/rheumatologist as the most reliable source of information about arthritis and expressed interest in identifying the following unknowns: o Discussing topics with their rheumatologist regarding their CVD risk in the setting of having IA o Questions they should ask about arthritis and CVD risk o The appropriate frequency of communication and visits with the rheumatologist o Better ways to engage their rheumatologist in addressing their concerns about medications, laboratory results, and symptoms
**Facilitator Theme**		
**Theme 5:*Potential for peer coaches (trained patients with IA) to help overcome barriers to screening and treatment of hyperlipidemia to lower CVD risk***	Peer/Social Support	Participants expressed interest in discussing the following topics with a peer coach (trained patient with IA): o How the peer coach managed having arthritis o Feelings about taking medications for arthritis o Benefits and issues that they have had with IA medications o Whether the peer coach has experienced a CVD event o What are they (peer coaches) doing to reduce their CVD risk
	Peer/Social Support Self-efficacy	o Best exercise program (weights, cardio, pool exercises) and location of related resources available in their local area o Having another patient with arthritis to engage with them in a workout program (workout partner) o Assistance in better communication with their doctor for adequate CVD screening and treatment

### Theme 1: need for more information about arthritis, prognosis, and IA medications prior to discussing additional topics like CVD risk

A dominant theme was the desire for patients to first understand their IA diagnosis before discussing other health risks such as CVD. Participants shared experiences of their initial diagnosis of IA. They expressed their concern about their initial lack of understanding about IA and knowledge about possible side effects of IA medications. Participants were primarily interested in understanding IA and wanted to learn what questions they, as patients, should proactively ask their doctors regarding prognosis of IA and side effects of medications for IA. They expressed issues of greatest concern to patients newly diagnosed with IA and suggested that rheumatologists could help by providing a list of these concerns to patients for consideration.

*“When I was first diagnosed, as far as the medications and the side effects and what I could expect.”*

*“They (rheumatologists) can put down there the most common questions asked (about arthritis). And then later, maybe put questions people don’t think to ask. You can’t be knowledgeable if you don’t know what to ask.”*

Across all focus groups, participants noted that gaining a better understanding of IA was a top priority. This was mainly because it could help them overcome fears about IA medications and cope with the stress of being diagnosed with IA.

### Theme 2: lack of knowledge about how IA increases CVD risk

Although many participants were aware of increased risk of CVD in IA, they expressed interest in learning more about how arthritis affects the heart and what they can do to decrease their CVD risk. Some of those who were unaware found this news shocking:

*“I never even thought about it. Had no idea that it would even affect my heart like that. I’m still in shock [laughter] that that has to do with the arthritis.”*

Some participants first learned about their CVD risk by participating in these focus groups. In fact, one participant felt motivated to request a cholesterol test from their doctor after learning about the importance of screening for hyperlipidemia:

*“I got a question for you. Since we are in this meeting and you feel like that cholesterol is linking to our RA, do you feel like we should ask for a cholesterol test?”*

Overall, *Theme 2* reflected a lack of knowledge about CVD risk among many participants of these focus groups, which stresses the importance of educating patients about this topic.

### Theme 3: lifestyle changes to reduce overall CVD risk rather than medications

As a CVD risk-reduction strategy, participants of this study expressed the importance of personal responsibility and voiced interest in diet, exercise, and stress reduction as ways to decrease CVD risk:

*“They (doctors) know about the medicines and everything, so I’ll ask them, ‘Is there something I could do or take that would help it?’ We’ve got to do our part, too. We’ve got to exercise. We got to watch what we eat. Stress. I know stress will cause a lot of stuff to come on.”*

Across all focus groups, participants expressed significant interest in engaging in lifestyle changes to lower their cholesterol level before initiating medications (e.g. statins). However, they were not familiar with the most effective way of making these changes and wanted more information about diet and exercise.

### Theme 4: improving doctor-patient communication about IA, medications, and CVD risk

Participants were interested in improving communication with their doctors, but they also emphasized that they trusted their doctors:

*“Furthermore, I feel like my doctor has my best—my health is in her best of interest. If she feels like I need a cholesterol test, she’ll give one, or recommending me someone that’s in that area. That’s how I feel about my doctor. I feel safe with her.”*

Another participant felt that communication is negatively affected when the doctor is in a rush:

*“When I feel like the doctor’s in a hurry … sometimes I feel like they’re in a hurry or they’re behind, or whatever. Then I get to where I don’t feel like I can really talk to them or have the time to talk to them and actually explain how I feel or what’s going on.”*

This participant expressed difficulty in communicating with one of their physicians in particular:

*“It’s like, first of all, I don’t care for him (doctor). [Laughter] He’s an older doctor and he’s like, ‘I’m only going to see you for one thing.’ I’m like, ‘No, if I have four things wrong with me, you need to see me for four things.’”*

Across focus groups, participants expressed that good communication with their doctor was important and positively affects their personal attitudes about their care. Participants stated that major barriers to good communication were limited time and the availability of their doctor to talk about their medical problems, including addressing health conditions that are CVD risk factors (e.g. hyperlipidemia). Participants across all focus groups stated that their rheumatologist did not discuss their increased CVD risk associated with having IA, which they thought was an important part of their care.

### Theme 5: potential for peer coaches (trained patient with IA in concepts of CVD and IA) to help overcome barriers to screening and treatment of hyperlipidemia to lower CVD risk

Participants across all focus groups, were not familiar with the concept of a peer coach, but once briefed on a peer coach’s role, participants discussed different activities for which they thought a peer coach would be useful regarding screening for hyperlipidemia. Below are the answers of several participants when asked about the ways a peer coach can help them communicate with their doctor regarding their cholesterol:

*“They (peer coaches) might be able to give you ways to say it (talk about cholesterol test), to where you can actually explain yourself to your doctor … how do I say that to her? Somebody to be able to kind of ease you into, okay, just talk about it like this (clearly to your doctor).”*

*“I know there was never a fasting cholesterol test, but if I had a peer person, then they might have mentioned it.”*

Participants expressed that a peer coach could also help them with aspects of IA beyond cholesterol testing. These included helping them overcome fears about a medication for IA, as stated by this participant:

*“It would’ve been helpful if I would talk to somebody who, maybe, was on the medication and could tell me, ‘Well, I haven’t had any problems with it,’ or, ‘Yeah, it does this.’ I’m sure it affects different people differently.”*

Others saw a peer coach as an exercise partner and as a source of encouragement.

*“I guess if it would involve exercising takin’ a walk, it’d be nice to do it with somebody if they’ve got the same (arthritis)—and do it together, that would be motivating.”*

*“For those that are going through whatever kind of hard time, they could really be helpful when you’re hitting a low time where you are like—can’t move and it hurts to walk. Those were devastating times for me. Just the encouragement.”*

Participants saw a peer coach as someone who could help them improve communication with their doctor.

*“The peer coach could go with you to your appointments and to help you to understand. I’ve thought about that, to completely understand and to help communicate.”*

*“If I’m at home, and all of a sudden, I’m like, ‘Why didn’t I ask her that? Let’s write it down.’ Then when I go to my peer coach, maybe, with those questions, they can also help me to understand that.”*

Participants across focus groups expressed interest in having a peer coach who is endorsed by their doctor, knowledgeable about arthritis, and has time available to work with them.

## Discussion

This study confirms past reports that many patients with IA are not adequately educated about their IA or about the CVD risk incurred by IA [35]. This group of patients also identified gaps in physician communication that contribute to this situation [36]. They expressed a desire to implement lifestyle changes to lower CVD risk, but expressed a need for more information about diet and exercise interventions. To our knowledge, this study is the first to highlight that patients with IA were receptive to working with a peer coach to get screened and treated for hyperlipidemia to lower their CVD risk.

Clearly, this study highlights the critical need for better education about IA as well as about CVD risk and risk reduction. Participants mentioned a sense of empowerment if they have a better understanding about CVD risk and were willing to engage in changes in diet and exercise. Education may not be enough for some patients, thus receptivity to working with a peer coach who is trained in behavior change is an important finding of this study. In our study, patients with IA were receptive to working with a peer coach as a vehicle to improve self-care, patient-doctor communication, and to obtain screening for hyperlipidemia *(Themes 3–5)*.

Several studies have shown similar results regarding patients’ lack of awareness of the association between IA and CVD risk. Bartels et al. conducted a series of interviews using grounded theory principles with 15 patients with RA, most of whom were unaware of this association [36]. Similarly, in another qualitative study of 14 patients with RA, Frølund et al. found that ten of 14 patients were unaware of the increased CVD risk among patients with RA [37]. In those studies, as observed in ours (*Theme 1)*, participants prioritized controlling their RA. It is very important not to overlook this fact because it will be unlikely that they will feel motivated to address CVD risk while still having active disease symptoms from IA. The participants in our focus groups expressed being relatively satisfied with their IA disease activity, which may explain why they felt more activated about requesting a laboratory test for cholesterol and engaging in lifestyle changes to improve their overall health and decrease CVD risk *(Themes 2 and 3)*. Nevertheless, this and past studies suggest that interventions to improve CVD risk in IA should include general information about IA and its treatments before focusing on CVD risk.

A recent study described that disclosing personalized risk for patients with RA was a motivation to modify behaviors like smoking cessation and flossing teeth [38]. In our study, we observed a similar pattern when patients felt motivated to request a cholesterol test after learning about their increased CVD risk during the focus groups discussions. Coaching patients to request a cholesterol test to increase screening for hyperlipidemia may be an appealing strategy, especially because many of these patients reported that their doctors did not discuss CVD risk with them.

Participants in our study welcomed the idea of working with a peer coach to motivate them to exercise, make dietary changes, help them communicate better with their doctor, and help them cope with the fears that they had regarding IA medications and their side effects *(Theme 5)*. Participants also envisioned peer coaches as a tool to help with motivation and self-management of IA. They thought that a peer coach could assist in not only managing IA, but to better understand CVD and as a facilitator to improve communication with their physician (*Theme 5*).

Several strengths of this study include the use of the SCT theoretical framework and a phenomenological study design, both of which allowed us to study the essential nature of how people living with IA understand their risk of CVD. The topic guide used in the focus groups was based on the constructs of this theoretical framework and was extensively vetted by investigators with different backgrounds and expertise, guaranteeing input from a variety of perspectives. We also achieved thematic saturation, which is the main goal of a qualitative study. The interpretation of the findings were aided by involvement of a patient with RA, enhancing patient-centeredness.

This study also has limitations. This qualitative study is hypothesis generating and the results may not be generalizable. Rather, this study identifies barriers and facilitators that can serve to inform possible ways where physicians and the healthcare system can intervene and address the low screening and poor treatment of hyperlipidemia that exists among patients with IA. Since we sampled individuals with IA who were 40 years or older, in accordance with ACC/AHA cholesterol treatment guideline CVD risk evaluation recommendation, this could have underrepresented the views of those younger than 40 years of age [14]. The patients in this study were from a single urban tertiary center, and it is possible that opinions will be different from individuals cared for in primary care settings. Participants were interviewed in focus groups, in which participation can be dominated by strong personalities and it is possible that more reserved participants did not have an opportunity to fully divulge their opinions. Finally, it is possible that the questionnaire could have both limited the possible reactions from participants as well as primed them for particular responses that the researchers were most interested in.

## Conclusions

The results of our study highlight the need to generate more effective patient-directed educational programming regarding IA, CVD risk such as hyperlipidemia screening and treatment, and CVD risk reduction strategies including lifestyle changes. It also highlights the continued need to improve doctor-patient communication. To our knowledge, it is  one of the first studies to suggest that some IA patients were receptive to working with trained peer coaches to both improve self-management behaviors and improve hyperlipidemia screening.

Patients with IA may need better education about IA in general and CVD risks, such as hyperlipidemia specifically. They expressed interest in lifestyle changes such as diet and exercise, but lacked sufficient information or guidance on how to engage in these changes. Interventions that use peer coaches seem to be of interest for patients with IA, and could help to activate and empower patients to receive screening and treatment for hyperlipidemia to enact behavioral changes to lower their risk.

## Supplementary information

**Additional file 1.** Topic guide for lipid lowering therapy focus groups.

## Data Availability

The datasets used and/or analyzed during the current study are available from the corresponding author on reasonable request.

## Abbreviations

IA: Inflammatory arthritis
CVD: Cardiovascular disease
RA: Rheumatoid arthritis
PsA: Psoriatic arthritis
EHR: Electronic health record
HIV: Human immunodeficiency virus
SCT: Social Cognitive Theory
UAB: University of Alabama at Birmingham
WCMC: Weill Cornell Medical College
