# Supplementary material for: Barriers and facilitators for screening and treatment of hyperlipidemia among patients with inflammatory arthritis
Source: BMC Rheumatol. 2020 Jun 2;4:26. doi: 10.1186/s41927-020-00123-w (PMC7265623; doi:10.1186/s41927-020-00123-w)
Supplement: Supplementary file 1 — Additional file 1. Topic guide for lipid lowering therapy focus groups. [file 41927_2020_123_MOESM1_ESM.docx]

**Supplement 1**

**Topic guide for lipid lowering therapy focus groups:**

*Welcome and thank you for agreeing to participate in this discussion. We are going to be talking about arthritis, heart attack, and stroke. In this discussion, there are no right or wrong answers and all thoughts are important. We do not want to miss your comments; therefore, the session will be recorded with your permission (audio). This discussion will last about 1 hour and you will receive $50.00 for your participation.*

**At this point:**

- **Participants will sign consent form and acknowledge audio recording form and make a name tag (we will indicate participants to create a name for confidentiality purposes and to not use their real name).**
- **Review ground rules**
- **Turn on audio recording**

Introduction (Participants will introduce themselves with their made up names to serve as an icebreaker for the discussion):

*Before we start, we want to tell you a bit about heart disease and stroke. You may already know that heart attack and strokes are most common cause of death in the United States, and they are also the most common cause of death among patients with RA, psoriatic arthritis, and ankylosing spondylitis. You may not know that the risk for heart attack and stroke are actually higher among patients with these types of arthritis compared to the general population. With that in mind, our conversation’s goals are:*

*First, we want to learn your perspectives on screening for heart disease and stroke risk, as well as managing this risk. We are particularly interested in your perspectives on cholesterol.*

*Second, we would like to discuss what kind of support you may wish to have to know more risk factors for cardiovascular disease and the things that you can do to reduce your risk. Specifically, we’ll talk about people with arthritis who might be trained as peer coaches. A peer coach is a person with arthritis who has had arthritis for a while, and is familiar with what is like to live with arthritis. Peer coaches are trained on how to help other people with RA live the fullest possible life. Peer coaches have been helpful to people who have other chronic illnesses like diabetes, but they have not been used as much for people with chronic arthritis. We’d like to hear your opinions about working with a peer coach.*

*Ok, let’s get started. I have told you that people with inflammatory arthritis like RA have increased risk for heart attacks and strokes. We know that there are risk factors for these conditions, and these risk factors include high blood pressure, diabetes, cigarette smoking, and high cholesterol. Today we’d like to focus on cholesterol. Treating cholesterol is a powerful way to decrease risks for heart attack and stroke, and there are very effective medications to treat cholesterol. In order to find out whether your cholesterol requires treatment, a blood test is needed, which is usually done at the doctor’s office, or at a lab with the doctor’s order.*

**Q. 1 Picture yourself discussing your risk for heart attack or stroke with your doctor. Now, let’s talk about how comfortable you are about talking with your doctor about heart attack and stroke risk. Would you be comfortable talking about this with your doctor?**

**Probe:**

- Why or why not?
- How comfortable are you talking with your PCP about your risks for heart attack and stroke? What about other issues related to your health?
- How comfortable are you talking with your rheumatologist about your risks for heart attack and stroke? How about other health related issues?
- Have you ever discussed getting tested and/or treated for high cholesterol with any of your doctor(s)? Why or why not?
- If you have discussed your risks, did you get tested for cholesterol? Why or why not?
- Who checked you for high cholesterol – was it your primary care doctor? Your rheumatologist? Anyone else?

**Q. 2 What kind of things makes you more or less comfortable talking with your doctor?**

1. **3 If you have a preference, who do you prefer to talk with about general health issues like risks for heart attack and stroke? Your rheumatologist? PCP? Other?**

**Probe**

- What are your thoughts about your rheumatologist addressing risk factors for heart attack and stroke such as cholesterol?
- Would you prefer that your rheumatologist addresses this at the same visit as for your arthritis? Why or why not?
- If you like the idea of your rheumatologist checking your cholesterol, how would you prefer it to be addressed? During the same visit as for your arthritis? At a separate visit? Why?

**Q. 4 What kind of conversations have you had with your doctor about checking you for cholesterol?**

**Probe:**

- Have you talked about this with your PCP?
- Have you talked about this with your rheumatologist? Any other doctor?
- Have you talked about this at all with any of your doctors? Why or why not?

**Q.4 In thinking about getting tested for high cholesterol, what are some of the things that might make it difficult for someone to get a blood test for cholesterol?**

**Probe:**

- What about who does the testing – is it more difficult if your primary care physician orders a cholesterol test, or your rheumatologist? If one is more difficult than the other, why is this? Is it because it’s harder to get the result from one or the other?
- What about how often you see each of these doctors? Do you see your rheumatologist more often, or your primary care doctor more often? Does how often you see the doctor affect how easy it is to get a blood test for you? Why or why not?
- How hard is it for you to talk with your doctor about getting a blood test done if your doctor isn’t the one who brought it up? Why do you think it’s hard? If it’s not hard, what makes it easy?
- How much of a problem is transportation to get a blood test done?
- How about work? Does your work schedule make it difficult to get a blood test?
- How about cost? Is the cost for a blood test a problem for you?
- Cholesterol tests are best done first thing in the morning before you eat. How much of a problem is the need to be fasting at the time of the blood test? If you forgot and were not fasting at the moment of blood collection, how much of a problem would it be to come back? How likely would you be to forget to come back?

**Q. 5 Let’s say that you’ve had your cholesterol measured and the doctor recommends a medicine to treat it. What are some of the things that you would like to know about the cholesterol medication before you decide that it is right for you?**

**Probe:**

- How much will it cost me?
- What are the side effects? How important is it to you to know how often any side effect happens? How mild, moderate, or severe the side effect is likely to be?
- How to take the medication?
- Interactions with other medications that you are taking? Specifically your RA medications?
- Do you think you are on too many pills already and don’t want to start a cholesterol medicine?
- What blood tests will you need to monitor while on this medication?
- How well this medication works? How much does it actually lower your risk?
- How you would know that it is working?
- How frequently you need to take this medication?
- How frequently you would need to visit the doctor because you are taking a medication for high cholesterol for monitoring?

**Q. 6 If your doctor recommended you to take a cholesterol medication, what are some the things that would may make it difficult for someone to take this medication?**

**Probe 1:**

- Cost?
- Fear of side effects?
- Not sure I need it?
- Frequency of labs needed while on this medication?
- Already taking too many medications?
- Are you concerned that you may forget to take it? Why?
- Are you experiencing side effects with other medications that you are already taking?
- How do you think this affects side effects for another medication? Is it related? How?

*We’ve just heard about a few things that people want to know before they decide whether a medicine like a cholesterol pill is right for them.*

**Q. 7 If we hadn’t had this discussion and you wanted to learn about the risks for heart attack and stroke, and how to reduce them with cholesterol medication, how would you prefer to learn about these things?**

***Probe:***

- From: Doctor? Nurse? Other health care provider?
- Friend? Family member? Other people taking the medicine? Other people with arthritis?
- At the doctors’ office? At home?
- How would you like to access this information? Online (internet)? Pamphlets? TV ads for medicine? Educational video?
- Other?

**Q. 8 If we were to create an educational video, what kind of things would make it something like that appealing to you?**

*Great. Thank you very much for sharing your opinions about risks for heart attack and stroke and cholesterol medication.*

*Now we would like to move on to our second goal, learning your perspectives on finding help and support. A lot of people find challenging or even overwhelming to work on risk reduction for heart disease while also managing a chronic illness like arthritis. Sometimes talking to a person like you who is facing similar challenges can be helpful. Peer coaches are people very similar to you in that they also have arthritis, but they have been trained to be supportive, to help you learn more about your own risk for heart disease and identify things that you can then use to reduce your own risk. They are not doctors or nurses or other health professionals. You might think of them like a personal trainer at a fitness center, only instead of helping you to become physically fit, they help you to live well with your arthritis. They can help you think or identify things that you may have not think about before that you will need to know and do to be and stay healthy. They provide emotional support, and help you deal with medications and how to better prepare for your doctors’ visits, in terms of questions that you should ask your doctor or even things that you can do to summarize and remember the things that your doctor is recommending for you. They typically talk with you in the phone or meet in person, often once a week or every couple of weeks.*

*Does anyone have questions about what a peer coach is?*

**Read this only if clarification is needed.** *A peer coach is a person who has had arthritis for a while and is familiar with the disease and its treatments. A peer coach has been trained on how to help other people with RA deal with their disease, medicines, and doctors.*

*Now that you know what a peer coach is,*

**Q. 9 What are your thoughts about working with a peer coach to help you reduce your risk for heart disease?**

**Probe:**

- Do you have any concerns about working with a peer coach? What are they?
- From your perspective, what are some advantages/benefits to working with a peer-coach?
- What are some potential disadvantages?
- What are your concerns in terms of your privacy while working with a peer-coach? How much of a concern is this for you?
- How willing would you be to work with a peer coach?
- How important is it to be able to pick your peer coach from a group of similarly trained individuals?

**Q. 10 What kind of activities would you want to do with a peer coach?**

- Talk on the phone? Meet in person to chat? Go for walks together?
- Go with you to the doctor? Discuss medicines? Discuss how to deal with your doctor better?
- Talk about how to handle friends and family who are not supportive of your flares?
- Talk about how to handle doctors who seem not to be interested in your fatigue or pain?

**Q. 11 If you were to work with a peer coach, how would you like to communicate with him/her?**

**Probe:**

- In person? Over the internet? Over the phone? Texting? E-mail? Skype?
- How important do you think it is to meet in person at least once?

*Let’s now move on to thinking about how a peer coach could help you with your interactions with your doctor. Earlier in our conversation we talked about how patients may feel comfortable or uncomfortable talking with their doctors, asking questions about their health risks and asking for tests the doctors have not brought up.*

**Q. 12 What kind of things could a peer coach do to manage those conversations easier?**

- **Help you prepare a list of things that you should ask?**
- **Role play, meaning, you rehearse with the peer coach how you will ask your doctors the questions that you have about your health and concerns about risk factors of your arthritis?**
- **Should a peer coach come with you to the visit?**
- **Others?**
